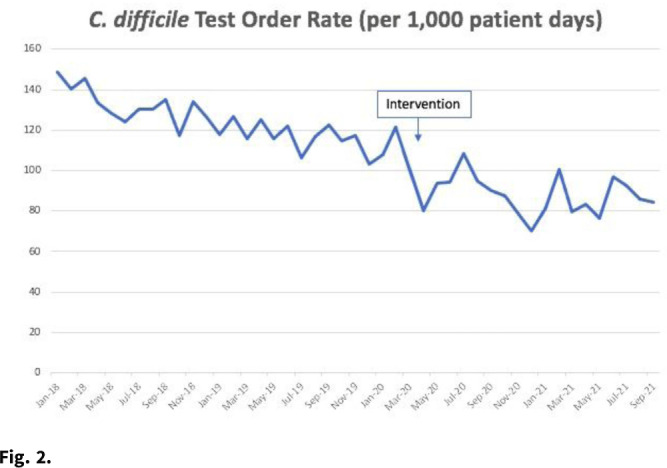# Role of diagnostic stewardship in reducing healthcare facility-onset *Clostridioides difficile* infections

**DOI:** 10.1017/ash.2022.108

**Published:** 2022-05-16

**Authors:** Anita Shallal, Medha Cherabuddi, Lance Podsiad, Christopher Gortat, Clare Shanahan, Jessica Kirkpatrick, Eman Chami, Stephanie Schuldt, Tarlisha Holsey, Martin Levesque, George Alangaden, Geehan Suleyman

## Abstract

**Background:** *Clostridioides difficile* infection (CDI) is the most common healthcare-associated infection (HAI) in the United States. Healthcare facility-onset (HO) CDI reporting is a laboratory-identified (LabID) event and does not rely on symptoms. Inappropriate testing can lead to overdiagnosis in patients who are colonized, especially in those receiving promotility agents. Approximately 45% of HO-CDI cases at our institution occurred in the setting of laxative use in 2019. We assessed the effectiveness of an electronic medical record (EMR) “hard stop” in reducing inappropriate CDI testing and its impact on HO-CDI rates. **Methods:** We conducted a pre–post quasi-experimental retrospective study comparing test order rates per 1,000 patient days, CDI rate per 1,000 patient days, and standardized infection ratio (SIR) in the preintervention period (January 2018–December 2019) to the intervention period (April 2020–September 2021), at a 5-hospital healthcare system in southeastern Michigan. In February 2020, we implemented a hard stop in Epic that was triggered >3 days after admission for the following criteria: patients <1 year of age; repeated testing within 7 days, and receipt of promotility agents within 48 hours. After discontinuing the promotility agents for at least 48 hours, providers were allowed to place an order if diarrhea persisted. The medical director of infection prevention and control or designee had the ability to override the hard stop when deemed necessary after reviewing the case upon provider request. All orders expired after 24 hours if a specimen was not collected. We retrospectively reviewed the number of overrides after the intervention to determine the positivity rate. **Results:** Our CDI rates per 1,000 patient days were 3.21 in the preintervention period and 1.48 in the postintervention period, a 54% reduction (Fig. 1). The test order rates were 119.4 in the preintervention period and 87.7 in the postintervention period, a 26.5% reduction (Fig. 2). The SIR decreased from 0.542 in the preintervention period to 0.361 in the postintervention period, a 33% reduction (95% CI, 0.54–0.82; *P* = .0001). After the intervention, 299 patients had an override. Of these, samples from 218 patients (72.9%) were negative, 50 orders (16.7%) were cancelled, and 28 samples (9%) were positive. **Conclusions:** Diagnostic stewardship, utilizing an electronic hard stop, was effective in reducing inappropriate *C. difficile* testing in the setting of promotility agents without delaying diagnosis of HO-CDI. This strategy combined with standard best practices can significantly reduce HO-CDI rates.

**Funding:** None

**Disclosures:** None

**Figure:**
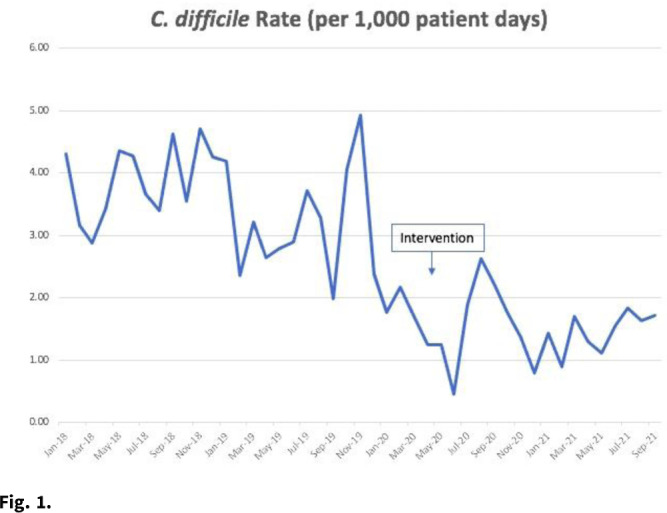

**Figure:**